# Hot Deformation Behavior of 7085 Aluminum Alloy Based on Constitutive Model, Processing Map, and Microstructure Evolution

**DOI:** 10.3390/ma19010091

**Published:** 2025-12-26

**Authors:** Wenke Wang, Wenqing Li, Xiaolong Tang, Yuehua Sun, Jian Ren

**Affiliations:** 1 HIT (Weihai) Innovation Park Corporation Limited, Weihai 264209, China; 15b309020@hit.edu.cn (W.W.); liwenqing_2020@163.com (W.L.); 2 School of Materials Science and Engineering, Anhui University of Technology, Maanshan 243002, China; txl000206@163.com (X.T.); sunyuehua1008@126.com (Y.S.)

**Keywords:** 7085 aluminum alloy, hot compression, processing map, constitutive model

## Abstract

To understand the hot deformation behavior of 7085 aluminum alloy, compression tests were performed under varied conditions (593–743 K/0.001–1 s^−1^). While the true stress–strain curves predominantly display the features of dynamic recovery, the softening mechanism shifts towards dynamic recrystallization when deforming at higher temperatures and lower strain rates. The validity of the constructed strain-compensated Zener–Hollomon model is confirmed by its exceptional precision in forecasting the flow stress, achieving an R^2^ value of 0.992. The instability areas are concentrated in the high-strain-rate regions, and the optimal deformation processing for 7085 aluminum alloy is 693–743 K/0.01–0.001 s^−1^. The alloy’s softening mechanism undergoes a transition from solely dynamic recovery to a progressively more significant coordinated role of dynamic recovery and dynamic recrystallization as the temperature rises and the strain rate drops.

## 1. Introduction

The status of 7085 aluminum alloy as a key aerospace material for large-scale integrated components (e.g., wrought ring products and aircraft frames) stems from its exceptional property profile. As an ultra-high strength alloy from the Al-Zn-Mg-Cu family, it offers an optimal balance of high specific strength, excellent hardenability, and good stress corrosion resistance [1,2,3]. However, this alloy faces considerable challenges during thermomechanical processing, particularly in terms of the ambiguous deformation–phase transformation coupling mechanisms and the difficulties in controlling microstructural homogeneity. These issues often lead to coarse-grain ring defects and mechanical property anisotropy in forgings, thereby limiting their applications under extreme service conditions [4,5]. In response to the aerospace industry’s demands for ultra-high strength aluminum alloys with high performance, integrality, large dimension, and complex geometry, breakthroughs must be achieved from two dimensions: material design and preparation technology. On the one hand, high-strength, high-toughness, and high-hardenability thick-section materials are developed through multi-component microalloying design. On the other hand, multi-scale characterization is employed to analyze the dynamic dissolution and precipitation behavior of second-phase particles, and a quantitative relationship model between the processing map and microstructural evolution is established. This provides a theoretical basis for optimizing the solution-aging process window, suppressing the formation of coarse-grain ring defects, and improving the overall service performance of components. While current research priorities focus on establishing constitutive models that incorporate dynamic recovery/recrystallization kinetics and precipitation thermodynamics, it is worth noting that advanced processing map construction methods integrating machine learning algorithms have shown advantages in determining optimal thermal working windows. Enhancing the precision manufacturing of next-generation aerospace components necessitates gaining deeper insights into the mechanisms of microstructural evolution and high-temperature deformation characteristics in 7085 aluminum alloy.

The flow stress and constitutive equation are central to understanding the hot deformation of aluminum alloys. This process is characterized by the competition between work hardening and dynamic softening, which are simultaneously influenced by deformation parameters (temperature, strain rate, and strain), initial microstructure, and alloy composition, leading to diverse flow behaviors. At the initial stage of deformation, the rapid increase in dislocation density leads to the dominance of work hardening. As deformation proceeds, enhanced thermal activation promotes the occurrence of dynamic softening. Aluminum alloys primarily undergo dynamic recovery as the main softening mechanism, where dislocation rearrangement and annihilation partially counteract the hardening effect; dynamic recrystallization may also occur at elevated temperatures [6]. Serving as a cornerstone for understanding thermomechanical processing, constitutive equations are routinely applied to map the hot working behavior of diverse metallic materials, from aluminum and magnesium alloys to titanium alloys and steels [7,8,9].

Mohammadi et al. [10] utilized the Zener–Hollomon parameter to characterize the flow stress of Zn–22Al alloy. An excellent agreement was observed between the model and the experiment, with a marginal deviation of 8.27%, demonstrating the equation’s high fidelity in modeling the alloy’s deformation at elevated temperatures. During high-temperature compression testing, the key parameters governing dynamic recovery and dynamic recrystallization include deformation temperature, strain rate, and strain. To gain deeper insights into the thermal deformation behavior and microstructure evolution of metals, numerous researchers have performed extensive studies utilizing processing maps developed using the Dynamic Material Model (DMM) framework. These studies aim to precisely characterize material behavior under hot working conditions and identify potential instability zones. Li et al. [11] investigated the hot deformation characteristics of 7050 aluminum alloy, focusing on its flow behavior and the evolution of its microstructure. Based on this, they optimized the hot deformation process using hot processing maps.

In this work, the microstructure evolution and softening mechanism of 7085 aluminum alloy during hot deformation at different temperatures and strain rates were investigated. A strain-compensated Arrhenius-type constitutive model was developed to characterize the high-temperature flow behavior of the 7085 aluminum alloy. The predictive performance and reliability of the model were further assessed using key statistical metrics, namely the mean absolute relative error (AARE), correlation coefficient (R), and root mean square error (RMSE). The causes of plastic instability and the optimal processing technology were determined by analyzing the hot processing maps and microstructure evolution.

## 2. Experimental Procedures

**Figure 1:**
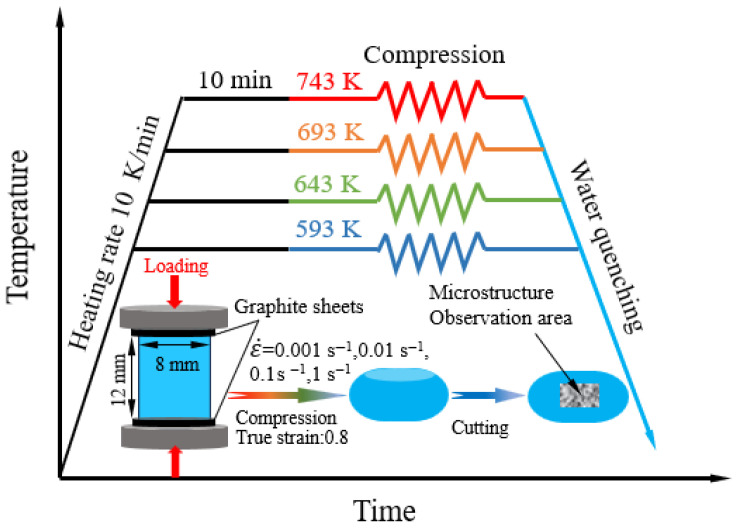
Schematic diagram of hot compression experimental process.

This study employed a commercial Al-7.3Zn-1.8Cu-1.1Mg-0.1Sc-0.1Zr alloy subjected to a homogenization treatment at 743 K for 8 h. The hot compression experiments were carried out on a Gleeble-3500 thermal–mechanical simulator. Cylindrical samples with a geometry of 8 mm diameter and 12 mm height were utilized. The experimental setup for hot compression is depicted schematically in Figure 1. The testing samples were heated to the preset test temperature at a heating rate of 10 K/min. After holding for 10 min, the testing samples were compressed to a true strain of 0.8 at the preset strain rate. Then, the compressed samples were quickly quenched in room-temperature water to maintain the deformed microstructure. In this experiment, the compression temperature range is 593–743 K, and the compression strain rate range is 0.001–1 s^−1^. To reduce interfacial friction during compression testing, the samples were prepared by grinding and polishing both end faces, and graphite sheets were used as interfaces between the prepared samples and the compression molds when installing the samples.

Following hot compression, the specimens were sectioned parallel to the compression direction to examine the microstructural characteristics at the center of the cross-section, as illustrated in Figure 1. The deformed samples were subjected to microstructural characterization using a polarizing optical microscope (OM, Soptop CX40P, Ningbo Shunyu Optical Company, Ningbo, China). For more detailed analysis, a Hitachi SU-5000 scanning electron microscope (SEM) (Hitachi, Tokyo, Japan) equipped with an electron backscatter diffraction (EBSD) detector and a JEM 2010 transmission electron microscope (TEM) (JEOL, Tokyo, Japan) was employed.

The samples for OM observation were first ground and mechanically polished, and then anodically coated using an etchant composed of 5 mL HBF_4_ and 200 mL H_2_O. The samples for EBSD testing were mechanically ground and then electrolytically polished using an electrolyte composed of 15% HClO_4_ + 85% CH_3_OH. The samples for TEM observation were first mechanically ground to a thickness of 100 μm, and then electrochemically thinned by double-jet polishing using an electrolyte composed of 25% HNO_3_ + 75% CH_3_OH until perforation. The specific parameters for double-jet polishing were as follows: a temperature of 243–253 K, an operating current of 50–60 mA, and an operating voltage of 10–15 V.

## 3. Results and Discussion

### 3.1. Flow Behavior of 7085 Aluminum Alloy

**Figure 2:**
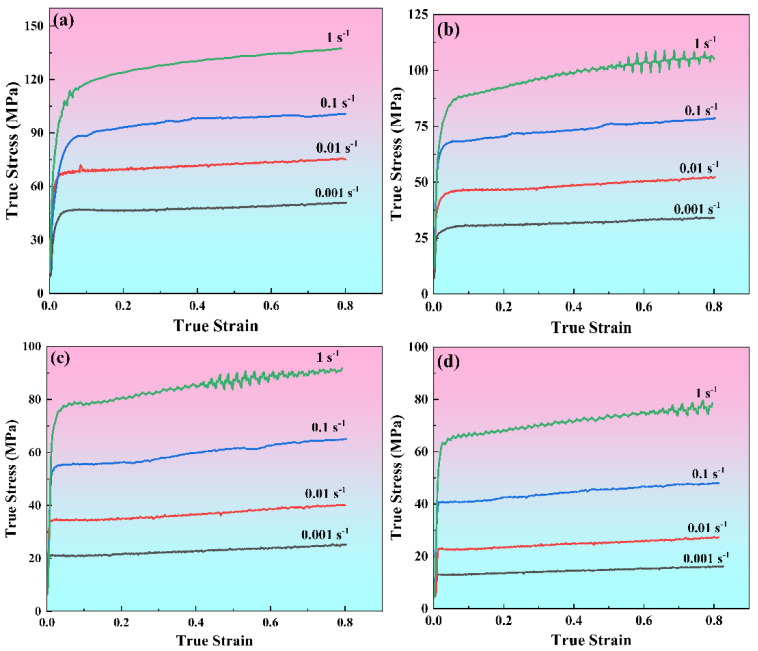
True stress–strain curves of 7085 alloy compressed under different deformation conditions: (**a**) 593 K, (**b**) 643 K, (**c**) 693 K, (**d**) 743 K.

The compressive behavior of the 7085 aluminum alloy under different thermal and strain rate conditions (593–743 K/0.001–1 s^−1^) is illustrated in Figure 2 through its true stress–strain curves. A similar trend is observed across all curves at the initial stage. That is, true stress rises sharply with the increase in true strain. This is because work hardening dominates deformation in the initial deformation stage due to the exponential growth of dislocation density [12]. After the work hardening stage is over, the true stress–strain curves of the alloy show some differences. At high strain rates (such as 1 s^−1^ and 0.1 s^−1^), true stress tends to increase slowly and fluctuate up and down as true strain accumulates continuously. This is chiefly attributed to the ongoing interplay of dynamic softening and work hardening during the hot compression process, wherein dynamic softening fails to fully negate the effects of work hardening [13]. As the strain rate decreases (such as 0.01 s^−1^ and 0.001 s^−1^), the true stress tends to stabilize or fluctuate slightly with the continuous accumulation of true strain, which indicates that dynamic softening and work hardening reach a dynamic equilibrium. The above-mentioned true stress–strain curves do not show obvious stress peaks, indicating that the alloy under these compression conditions exhibits typical dynamic recovery (DRV) characteristics; that is, dynamic softening is dominated by DRV. In addition, at certain high temperatures and low strain rates (693 K/0.001 s^−1^, 743 K/0.01–0.001 s^−1^), there are stress peaks on the true stress–strain curves, indicating that dynamic recrystallization (DRX) is also involved in the dynamic softening process of 7085 aluminum alloy. Notably, Figure 2 reveals an inverse correlation of true stress with deformation temperature, alongside a direct dependence on strain rate. Specifically, the reduced strain rates combined with the elevated deformation temperatures promote dislocation rearrangement through two synergistic effects [6]: (1) enhanced thermal activation providing adequate driving force for dislocation annihilation, and (2) prolonged deformation duration facilitating dislocation redistribution processes. Consequently, these conditions are conducive to intensified dynamic softening rather than work hardening effect [14].

### 3.2. Establishment and Verification of Constitutive Model for 7085 Aluminum Alloy

The flow behavior of Al-Zn-Mg-Cu alloys is frequently modeled using an Arrhenius-type constitutive equation. This model incorporates the Zener–Hollomon parameter (Z) and establishes the correlation between flow stress, deformation temperature, and strain rate across various stress levels [15]. The specific formula is expressed as follows:

(1) $$Z=\dot{\varepsilon }\exp\left(\frac{Q}{RT}\right)=\{\begin{array}{cc} A_{1}\sigma^{n_{1}} & \left(\alpha \sigma <0.8\right)\\ A_{2}\left[exp\left(\beta \sigma \right)\right] & \left(\alpha \sigma <1.2\right)\\ {A[sinh(\alpha \sigma )]}^{n} & \left(\mathrm{for}\;\mathrm{all}\;\sigma \right) \end{array}$$

Here, the strain rate (s^−1^) and true stress (MPa) are symbolized by $\dot{\varepsilon }$ and *σ*, respectively. The model also incorporates the activation energy for hot deformation, *Q* (kJ/mol), the universal gas constant *R* (8.314 J·mol^−1^·K^−1^), and the absolute temperature *T* (K). Additionally, the terms *A*, *A*_1_, *A*_2_, *n*_1_, *n*, *β*, and *α* (with *α* defined as *β*/*n*_1_) are material constants.

Taking the natural logarithm of Equation (1), the following equation [16] is obtained:

(2) $$lnZ=ln\dot{\varepsilon }+\frac{Q}{RT}=\{\begin{array}{cc} lnA_{1}+n_{1}ln\sigma & \left(\alpha \sigma <0.8\right)\\ {lnA}_{2}+\beta \sigma & \left(\alpha \sigma <1.2\right)\\ lnA+nln[sinh(\alpha \sigma )] & \left(\mathrm{for}\;\mathrm{all}\;\sigma \right) \end{array}$$

Assuming that *Q* is not a function of *T*, then linear relationships exist between $ln\dot{\varepsilon }$ and lnσ, $ln\dot{\varepsilon }$ and σ, and $ln\dot{\varepsilon }$ and ln[sinh(ασ)]. $n_{1}$, β, and n correspond to the slopes in these linear relationships and are represented by the following equations.

(3) $$n_{1}={\frac{\partial ln\dot{\varepsilon }}{\partial ln\sigma }|}_{T}$$

(4) $$\beta ={\frac{\partial ln\dot{\varepsilon }}{\partial \sigma }|}_{T}$$

(5) $$n={\frac{\partial ln\dot{\varepsilon }}{\partial ln\left[sinh\left(\alpha \sigma \right)\right]}|}_{T}$$

Assuming that $\dot{\varepsilon }$ is a constant, then ln[sinh(ασ)] and $\frac{1}{T}$ exhibits a linear relationship and $\frac{Q}{Rn}$ is the slope of this linear relationship. Therefore, *Q* can be calculated by Equation (6).

(6) $$Q=R\cdot n{\cdot \frac{\partial ln\left[sinh\left(\alpha \sigma \right)\right]}{\partial \left(\frac{1}{T}\right)}|}_{\dot{\varepsilon }}=R\cdot n\cdot k$$

**Figure 3:**
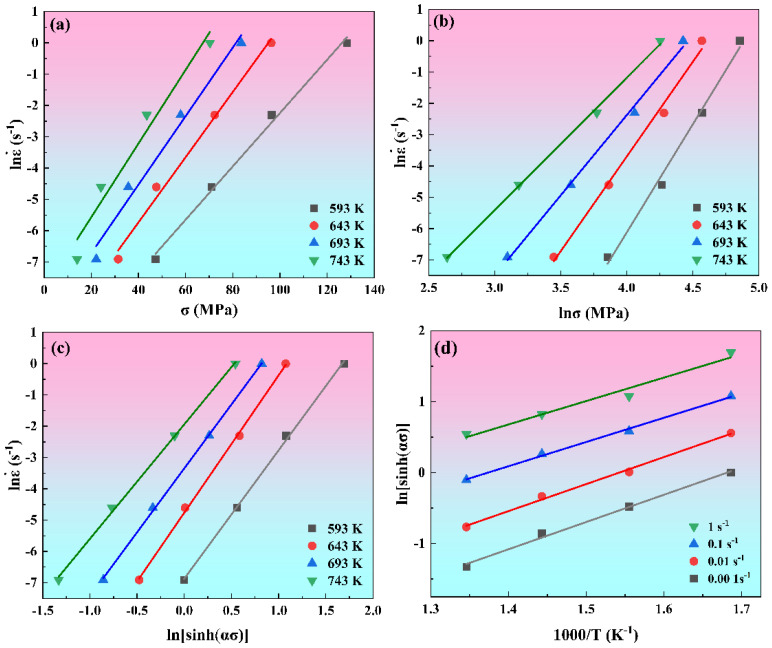
The linear relationship between (**a**) $\ln\dot{\varepsilon }$ and σ, (**b**) $\ln\dot{\varepsilon }$ and lnσ, (**c**) $\ln\dot{\varepsilon }$ and lnlnsinh(ασ), (**d**) lnsinh(ασ) and 1000/T.

To demonstrate the methodology for determining these material constants, a strain of 0.3 is selected as a case study. Figure 3 displays the linear relationships between (a) ln$\dot{\varepsilon }$ and σ, (b) ln$\dot{\varepsilon }$ and lnσ, (c) ln$\dot{\varepsilon }$ and ln[sinh(ασ)], and (d) ln[sinh(ασ)] and 1000/*T* at strain of 0.3. According to Equations (3)–(6), performing linear fitting on the data in Figure 3 and taking the average slopes to evaluate the values of β, $n_{1}$, n, and *k*, which are 0.1041, 5.5874, 4.5084, and 3.5895, respectively. Therefore, the values of α and Q at a strain of 0.3 can be calculated as 0.0187 and 121.06 kJ/mol, respectively.

**Figure 4:**
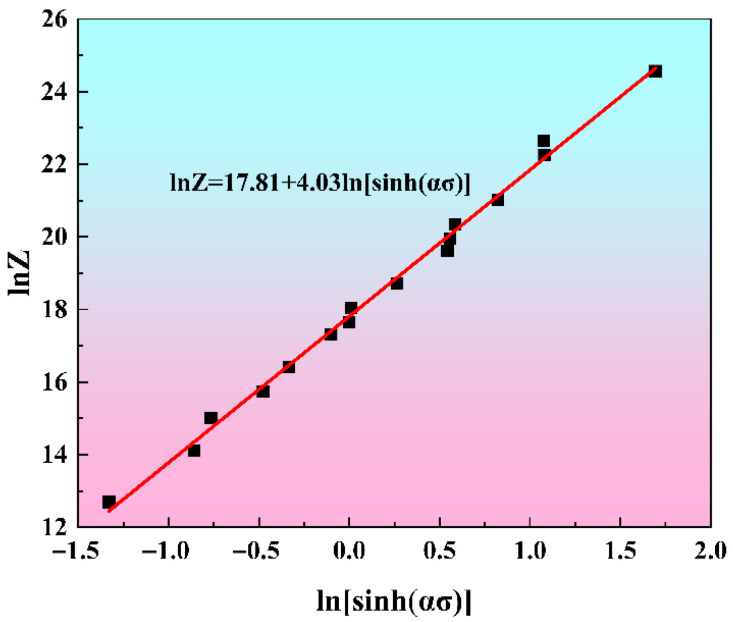
The linear relationship between lnZ and ln[sinh(ασ)] at a strain of 0.3.

According to Equation (1), for a given strain, 12 values of *Z* can be calculated by using different compression experimental parameters ($\dot{\varepsilon }$ and *T*) and the calculated Q value. As illustrated in Equation (2), a linear correlation exists between ln*Z* and ln[sinh(*ασ*)], with ln*A* and n representing the intercept and slope, respectively. This relationship at a strain of 0.3 is presented in Figure 4. A linear regression fit to the data yields a slope of 4.03 and an intercept of 17.81, which correspond to the values of *n* and ln*A* at this specific strain.

**Figure 5:**
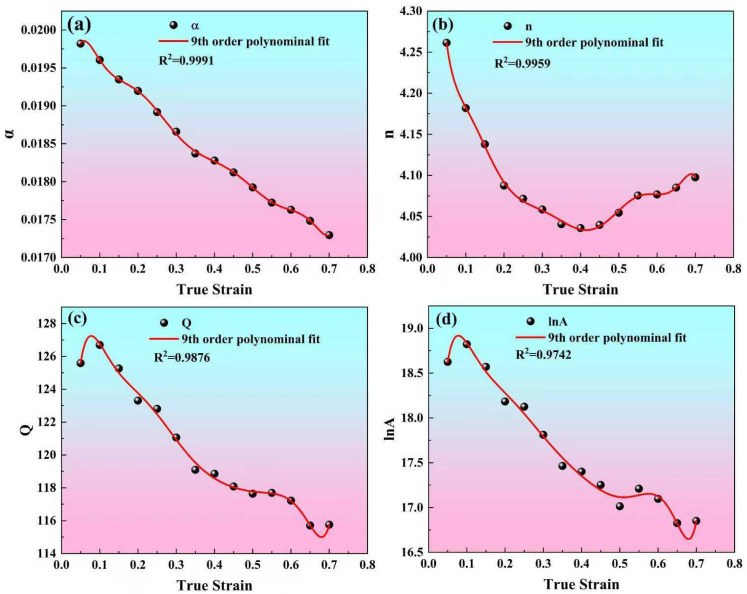
Ninth-order polynomial fitting curves for true strain and material parameters, including (**a**) α, (**b**) n, (**c**) Q, and (**d**) lnA.

As established in Equation (1), the flow stress (*σ*) exhibits a dependence on the parameters of the compression experiment, namely the strain rate ($\dot{\varepsilon }$) and temperature (*T*), as shown in Equation (7). That is, knowing the values of α, *n*, *A*, and Q, the flow stress can be calculated at a given deformation and strain rate. However, there are differences in the calculated values of α, *n*, *A*, and Q under different strains, indicating that these parameters are strain-dependent. In order to make the calculated flow stress closer to the experimentally measured values, strain is incorporated as a key variable alongside deformation temperature and strain rate, thereby establishing a strain-compensated Zener–Hollomon model. Within the true strain range of 0.1 to 0.8, the above calculations shown in Figure 3 and Figure 4 are repeated with a strain interval of 0.05, thereby obtaining the values of α, *n*, *A*, and Q, corresponding to different strains, as shown in Figure 5. Polynomial fitting is performed on the data points in Figure 5, and it is found that a ninth-order polynomial can well express the relationships between true strain and the material coefficients (α, *n*, *A*, and Q). The ninth-order polynomial is shown in Equation (8), in which $Y_{\left(\varepsilon \right)}$ is the material coefficients (α, *n*, *A*, and Q), and the fitted polynomial coefficients are presented in Table 1. Therefore, as presented in Equation (9), the flow stress can be formulated and precisely determined using the specified key processing parameters: deformation temperature, strain rate, and strain.

(7) $$\sigma =\frac{1}{\alpha }ln\left[{\left(\frac{Z}{A}\right)}^{\frac{1}{n}}+\sqrt{{\left(\frac{Z}{A}\right)}^{\frac{2}{n}}+1}\right]=\frac{1}{\alpha }ln\left[{\left(\frac{\dot{\varepsilon }exp(\frac{Q}{RT})}{A}\right)}^{\frac{1}{n}}+\sqrt{{\left(\frac{\dot{\varepsilon }exp(\frac{Q}{RT})}{A}\right)}^{\frac{2}{n}}+1}\right]$$

(8) $$Y_{\left(\varepsilon \right)}=B_{0}+B_{1}\varepsilon^{1}++B_{2}\varepsilon^{2}+B_{3}\varepsilon^{3}+B_{4}\varepsilon^{4}+B_{5}\varepsilon^{5}+B_{6}\varepsilon^{6}+B_{7}\varepsilon^{7}+B_{8}\varepsilon^{8}+B_{9}\varepsilon^{9}$$

(9) $$\sigma =\frac{1}{\alpha_{\left(\varepsilon \right)}}ln\left[{\left(\frac{\dot{\varepsilon }exp(\frac{Q_{\left(\varepsilon \right)}}{RT})}{A_{\left(\varepsilon \right)}}\right)}^{\frac{1}{n_{\left(\varepsilon \right)}}}+\sqrt{{\left(\frac{\dot{\varepsilon }exp(\frac{Q_{\left(\varepsilon \right)}}{RT})}{A_{\left(\varepsilon \right)}}\right)}^{\frac{2}{n_{\left(\varepsilon \right)}}}+1}\right]$$

**Figure 6:**
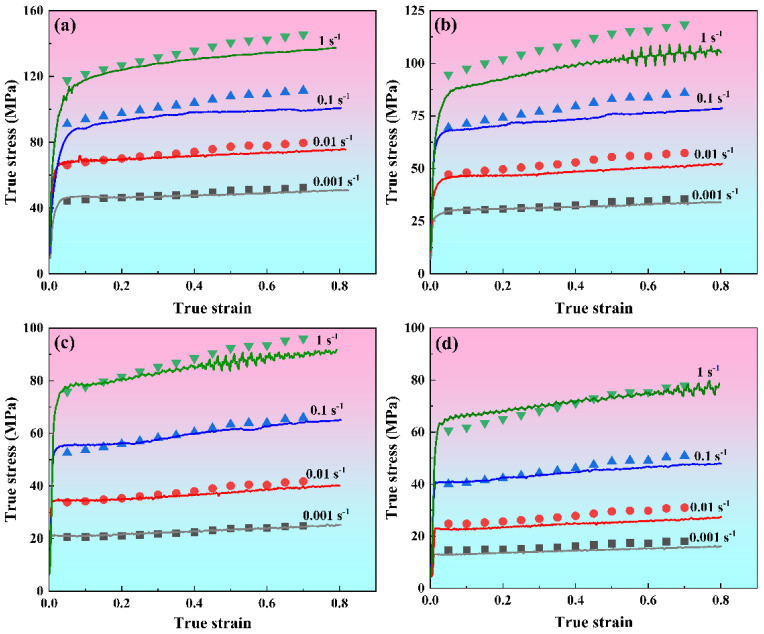
Comparison of experimental and predicted flow stress by using strain-compensated Zener–Hollomon model: (**a**) 593 K, (**b**) 643 K, (**c**) 693 K, (**d**) 743 K.

**Figure 7:**
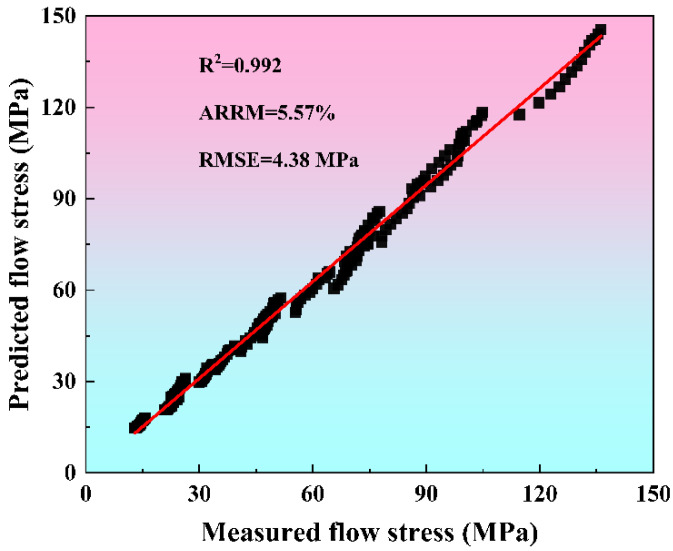
Correlation between experimental and predicted flow stress.

To evaluate the predictive capability of the strain-compensated Zener–Hollomon model, the flow stresses computed from Equation (9) are compared against experimental measurements in Figure 6. We substituted the polynomials of $\alpha_{\left(\varepsilon \right)}$, $Q_{\left(\varepsilon \right)}$, $A_{\left(\varepsilon \right)}$, and $n_{\left(\varepsilon \right)},$ expressed by ε into Equation (9). Given the *T*, $\dot{\varepsilon }$, and ε, the corresponding predicted stress values can be obtained. Figure 6 is obtained by superimposing the calculated predicted stress values with the measured values shown in Figure 2. The results indicate strong alignment between the model’s predictions and the experimental data, particularly at high temperatures and low strain rates. Conversely, at lower temperatures combined with higher strain rates, minor deviations are observed, which are mainly attributed to the linear regression method used to determine the material constants. The predictive accuracy of the model regarding flow stress is further assessed through statistical metrics, including the mean absolute relative error (AARE), correlation coefficient (R), and root mean square error (RMSE) [17]. As illustrated in Figure 7, a strong linear correlation exists between the experimentally obtained and model-predicted flow stress data. The calculated R^2^, AARE, and RMSE values are 0.992, 5.57%, and 4.38 MPa, respectively. These results further confirm the good predictive performance of the strain-compensated Zener–Hollomon model in estimating the flow stress behavior of 7085 aluminum alloy.

### 3.3. Establishment and Analysis of DMM Processing Map for 7085 Aluminum Alloy

The processing map based on a dynamic material model (DMM), combined with a power dissipation diagram and an instability diagram, has been widely used to study the relationship between thermal processing performance of alloys and processing parameters ($\dot{\varepsilon }$ and *T*), so as to determine the optimal thermal processing parameters of alloys [18,19,20]. The efficiency with which a material dissipates power can be evaluated using the parameter of power dissipation efficiency (*η*), defined as follows:

(10) $$\eta =\frac{J}{J_{max}}=\frac{2m}{m+1}$$

(11) $$m=\frac{\partial (\mathrm{lg}\sigma )}{\partial \mathrm{lg}\dot{\varepsilon }}$$

Here, *J* denotes the power dissipated during microstructural evolution, while *m* indicates the strain rate sensitivity. In general, a higher *η* value is typically associated with improved material formability. This parameter is a function of both the deformation temperature (*T*) and the applied strain rate ($\dot{\varepsilon }$). The two-dimensional contour plot that graphically represents this relationship is defined as the power dissipation map.

The application of the maximum entropy principle, in conjunction with a key postulate of irreversible thermodynamics (the extremum principle), allows for the derivation of an instability criterion. This criterion serves to eliminate processing parameters that induce material instability and is given by the following equation:

(12) $$\xi \left(\dot{\varepsilon }\right)=\frac{\partial ln(\frac{m}{m+1})}{\partial ln\dot{\varepsilon }}+m<0$$

where *ξ* is termed the instability parameter. Flow instability is likely to occur when *ξ* assumes a negative value. The instability parameter *ξ* is intrinsically dependent on the deformation temperature and the strain rate. Finally, the DMM processing map is constructed by integrating the flow instability map with the power dissipation map.

**Figure 8:**
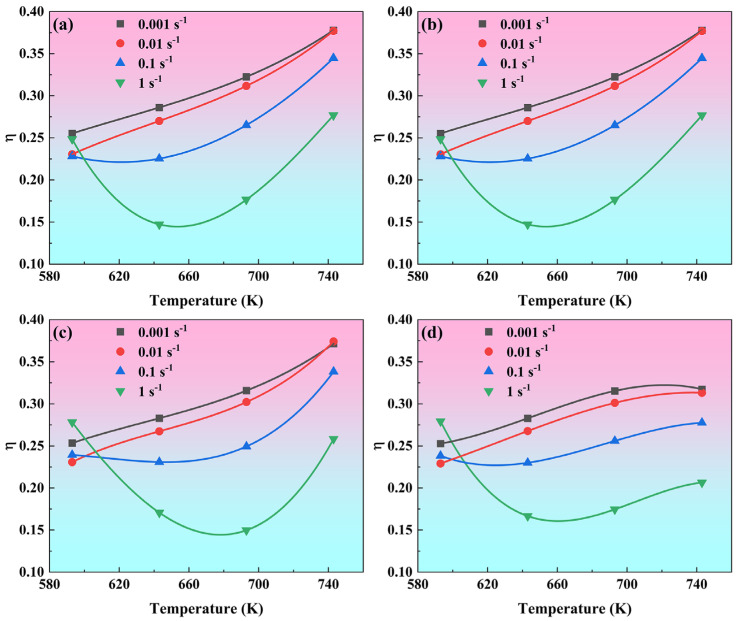
The power dissipation efficiency (*η*) as a function of deformation temperature and strain rate for four true strain levels: (**a**) 0.5, (**b**) 0.6, (**c**) 0.7, (**d**) 0.8.

Figure 8 displays the variation in power dissipation efficiency (η) in response to changes in deformation temperature and strain rate, evaluated at four true strain levels. The variation law of *η* value with temperature is almost the same at strains of 0.5, 0.6, and 0.7. As the temperature increases, *η* value shows a monotonically increasing trend for low strain rates of 0.01 s^−1^ and 0.001 s^−1^, *η* value first shows a slight decrease and then a rapid increase for a strain rate of 0.1 s^−1^, and *η* value first decreases significantly and then increases sharply. At a strain of 0.8, the variation in the η value with temperature at low temperatures is consistent with the law at low strain, but the *η* value trends to stabilize when the temperature is higher than 693 K. The *η* parameter exhibits a positive correlation with temperature and a negative correlation with strain rate. Specifically, when the strain is 0.8, the *η* value is observed to be marginally above 0.3 within the temperature and strain rate range of 693–743 K and 0.01–0.001 s^−1^, respectively.

**Figure 9:**
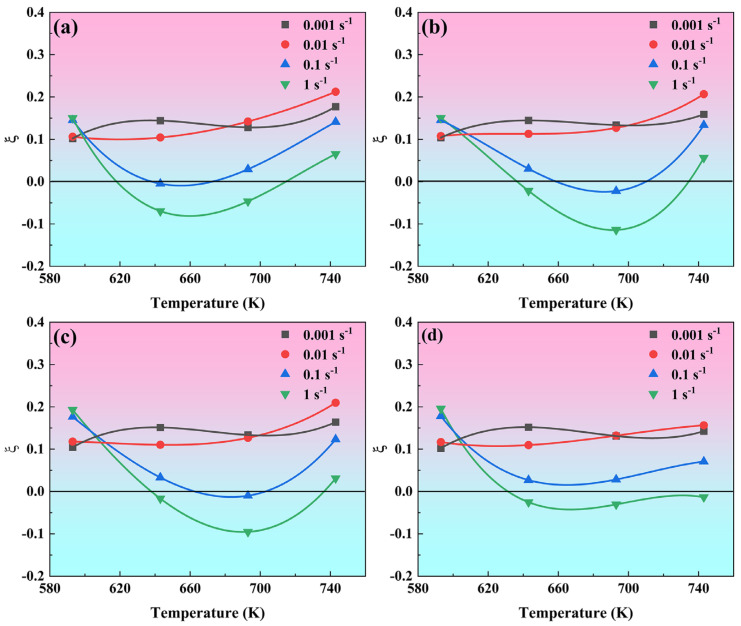
The flow instability parameter (ξ) as a function of deformation temperature and strain rate for four true strain levels: (**a**) 0.5, (**b**) 0.6, (**c**) 0.7, (**d**) 0.8.

Figure 9 illustrates the flow instability parameter (ξ) as a function of deformation temperature and strain rate at four true strain levels. At low strain rates (0.01 s^−1^ and 0.001 s^−1^), ξ value remains positive throughout the entire deformation temperature range, meaning that the material is in a stable flow state. When the strain rate increases to 0.1 s^−1^ and 1 s^−1^, ξ value first decreases and then increases with increasing temperature, and ξ value turns negative in all cases except at a strain of 0.8. It indicates that the influence of strain rate on the flow stability of the alloy is stronger than that of temperature, and plastic deformation at a low strain rate can avoid plastic instability.

**Figure 10:**
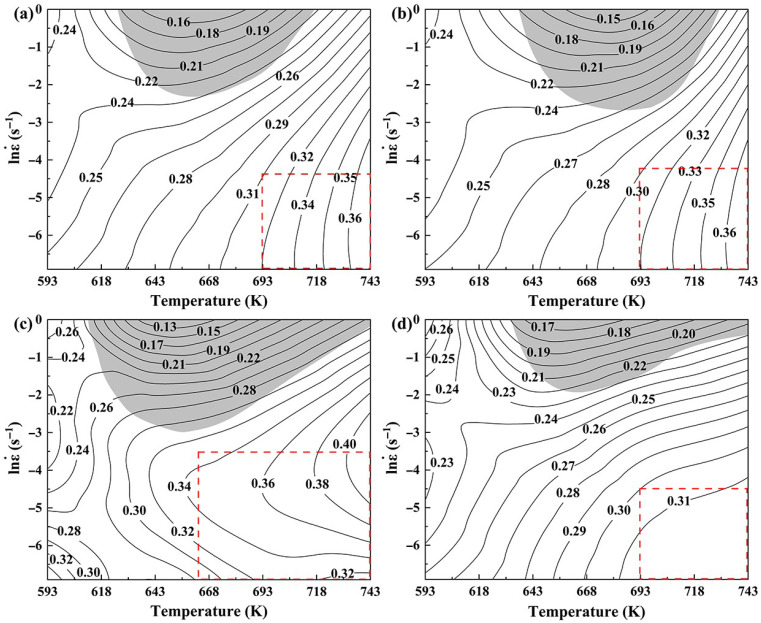
DMM processing maps of 7085 aluminum alloy under different strains: (**a**) 0.5, (**b**) 0.6, (**c**) 0.7, (**d**) 0.8.

Figure 10 shows the DMM processing map of 7085 aluminum alloy under true strains of 0.5, 0.6, 0.7, and 0.8. The numerical values on the contours correspond to the power dissipation efficiency (*η*). The gray shading delineates the instability region, identified by a negative value of the instability parameter (*ξ* < 0), in contrast to the adjacent white stability region. It can be seen that the area of the instability region first increases and then decreases as the strain increases, and the quantity always remains at one. The instability regions are located in the upper areas of processing maps, which correspond to high-strain-rate zones with lower power dissipation efficiency (0.13–0.28). It is widely recognized that materials are prone to flow instability at high strain rates, leading to the formation of adiabatic shear bands (ASBs), especially at low temperature and high strain rate [21]. In addition, the power dissipation efficiency is considerably lower in the instability zones, which is because the unstable material flow and the development of ASBs divert a large portion of the energy, dissipating it as heat instead of promoting microstructural evolution. Therefore, these instability regions are unsuitable for hot processing. Generally, a higher value of *η* is more favorable for plastic deformation. The *η* values corresponding to dynamic recovery, dynamic recrystallization, and superplasticity of aluminum alloys are 0.2–0.3, 0.3–0.6, and >0.6, respectively [22]. Usually, the processing parameters of alloys are selected in the region where the *η* value is higher than 0.3. The deformation mechanism of alloys is notably influenced by the density of contour lines. High-density contour lines indicate that the *η* value is more sensitive to the changes in deformation parameters, which is not conducive to controlling the microstructure of alloys in actual production. Therefore, the optimal processing window for alloys should be selected in the regions with *η* > 0.3 and sparse contour lines, as shown in the areas marked by a red dashed box in Figure 10. For 7085 aluminum alloy, the optimal processing window region is 693–743 K/0.01–0.001 s^−1^ at the true strain of 0.8.

### 3.4. Microstructure Evolution of 7085 Aluminum Alloy

**Figure 11:**
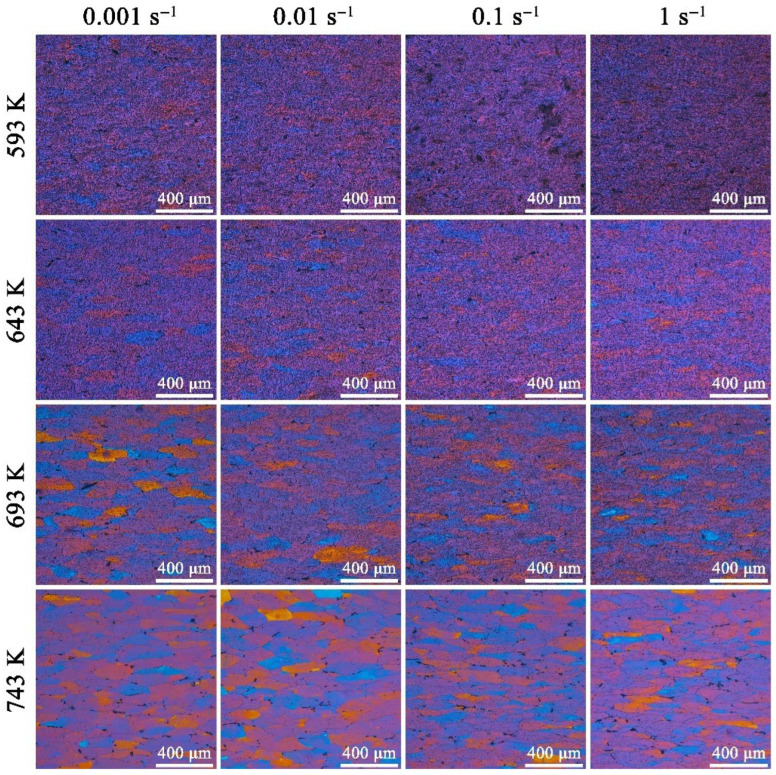
OM images of 7085 aluminum alloys compressed under different temperatures and strain rates.

Figure 11 shows the color OM image of 7085 aluminum alloy compressed under different temperatures and strain rates. When compressed at 593 K, the grain boundaries of the alloy are very blurred, and it is difficult to distinguish the complete grains from the OM images. As the temperature rises, the grain boundaries gradually become clearly visible, and the color of the grains becomes more prominent. The temperature reaches 743 K, and each grain can be distinguished from the OM images, especially at low strain rates. The distinctness of grain boundaries is primarily determined by the deformation temperature, with the strain rate playing a relatively minor role [23]. Overall, as the temperature rises and the strain rate decreases, the grain size of the alloy remains fairly stable, and the overall appearance of the grains shows little noticeable variation. When the samples are compressed at low temperatures and high strain rates, the grains are flattened, a large number of dislocations are generated within the grains, and dislocation movement leads to dislocation entanglement and accumulation at the grain boundaries. A large number of dislocations and the overlapping strain fields around them make the OM images appear overly noisy, with chaotic background contrast, which will mask the main macroscopic structure information, such as grain boundaries and phase boundaries, leading to a decrease in image clarity. With elevated temperatures and a reduced strain rate, the dislocations generated by work hardening in the alloy undergo heteromorphic cancelation, climbing, and rearrangement. This leads to dynamic recovery and dynamic recrystallization [24], resulting in a uniform and single coating. Consequently, the OM images exhibit clear grain boundaries and bright color.

**Figure 12:**
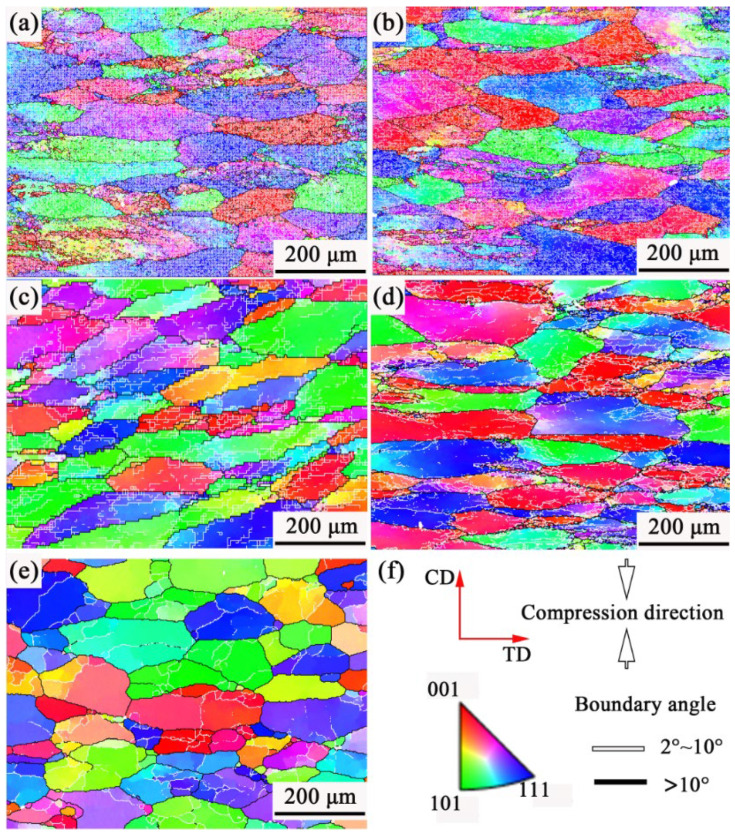
(**a**–**e**) EBSD images and (**f**) inverse pole figure (IPF) color code of 7085 aluminum alloy compressed under different conditions: (**a**) 593 K/1 s^−1^, (**b**) 643 K/1 s^−1^, (**c**) 693 K/1 s^−1^, (**d**) 693 K/0.1 s^−1^, (**e**) 743 K/0.001 s^−1^.

**Figure 13:**
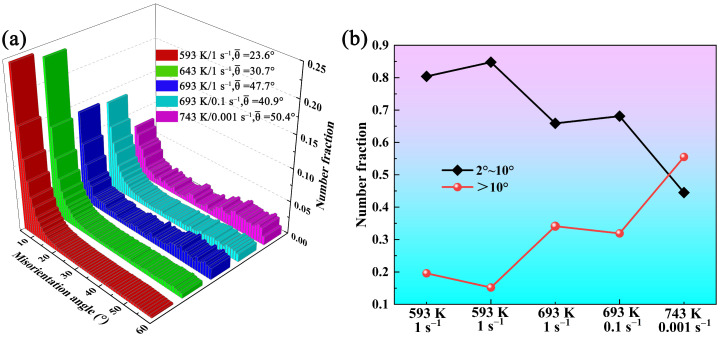
(**a**) Gain boundary misorientation distribution and (**b**) fractions of grain boundaries with different misorientation angles of 7085 aluminum alloys compressed under different conditions.

Figure 12 displays the EBSD images of 7085 aluminum alloy compressed under different conditions. At 593 K/1 s^−1^, the alloy microstructure is composed of a large number of coarse and flattened grains with blurred grain boundaries, and the grains are filled with low-angle grain boundaries (LAGBs, 2–10°, marked as white lines). This is also the reason why the grain boundaries are blurred, and the images are noisy in the color OM images under low-temperature compression conditions, as shown in Figure 11. Compared with the microstructure compressed at 593 K/1 s^−1^, the grain state in the alloy compressed at 643 K/1 s^−1^ is similar; the grains are characterized by a high prevalence of low-angle boundaries, and there is local inhomogeneity in their distribution. As the temperature rises to 693 K (Figure 12c), the density of subgrain boundaries in the alloy significantly decreases, and the grain boundaries of the original coarse grains show obvious serrated shapes. At 693 K/0.1 s^−1^, many fine dynamic recrystallized grains can be seen at the grain boundaries of the original coarse grains. When the temperature increases to 743 K, and the strain rate decreases to 0.001 s^−1^, the original grains are no longer in a very flattened state; obvious subgrains can be observed within the coarse grains, and larger recrystallized grains can be seen clearly at the grain boundaries. Figure 13 presents the grain boundary misorientation distribution and the boundary fractions with varying misorientation angles for 7085 aluminum alloy under different compression conditions. It shows that the average misorientation angle increases first and then decreases as the temperature increases and the strain rate decreases. When the samples are compressed at 593 K/1 s^−1^, 643 K/1 s^−1^, 693 K/1 s^−1^, 693 K/0.1 s^−1^, and 743 K/0.001 s^−1^, the corresponding ratios of low-angle to high-angle grain boundaries (HAGBs, > 10°, marked as black lines) are 0.804 and 0.196, 0.848 and 0.152, 0.659 and 0.341, 0.681 and 0.319, and 0.445 and 0.555. It can also be clearly seen from the statistical data that as the temperature rises and the strain rate decreases, the alloy state gradually changes from the dominance of work hardening caused by high-density dislocations to the dominance of softening dominated by dynamic recovery and dynamic recrystallization [25]. The true stress–strain curve for the alloy in Figure 2 confirms this behavior: under low-temperature and high-strain-rate conditions, the stress increases progressively until a true strain of 0.8 is attained. Conversely, when deformed at high temperatures and low strain rates, the stress plateaus after a certain strain level.

**Figure 14:**
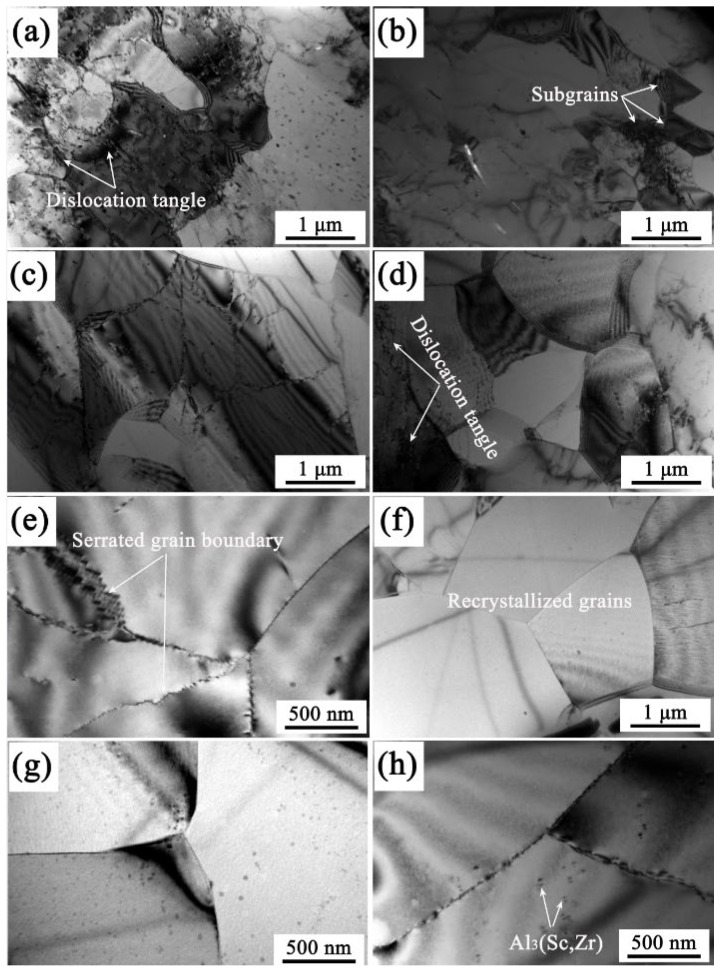
TEM images of 7085 aluminum alloys compressed under different conditions: (**a**,**b**) 593 K/1 s^−1^, (**c**,**d**) 693 K/1 s^−1^, (**e**,**f**) 693 K/0.1 s^−1^, (**g**,**h**) 743 K/0.001 s^−1^.

Figure 14 presents the TEM images of 7085 aluminum alloy compressed under different conditions. At 593 K/1 s^−1^, as shown in Figure 14a,b, the microstructure contains a large number of dislocations, and dislocation entanglement occurs, and a small number of subgrains can also be observed. When the temperature rises to 693 K (Figure 14c,d), the dislocation density within grains has decreased, severe dislocation entanglement still exists in local areas, and a large number of dislocation cells and a small amount of recrystallized grains have appeared. At 693 K/0.1 s^−1^, as shown in Figure 14e,f, many serrated grain boundaries and recrystallized grains are observed in the microstructure. During the hot deformation process, a large number of dislocations are generated within grains due to plastic deformation, forming high-energy deformed structures (such as dislocation entanglement and cellular structures). However, due to the need for coordinated deformation, the dislocation density at grain boundaries and their adjacent regions is usually low [26]. At this point, a huge energy storage difference is formed between the high-strain grains (with high energy) and the low-strain regions near the grain boundaries (with low energy) [27]. This kind of serrated grain boundary is prone to occur at moderate temperatures, where dynamic recovery is fully carried out, accumulating sufficient driving force to drive grain boundary migration, but not enough to trigger complete recrystallization, thus forming serrated grain boundaries [28]. When the temperature rises to 743 K, and the strain rate drops to 0.001 s^−1^, the dislocation density within grains is very low, and recrystallized grains with clear and straight grain boundaries grow. In addition, there are some subgrains with irregular grain boundaries, and horseshoe-shaped Al_3_(Sc,Zr) particles are also observed within the grains. At higher temperatures and lower strain rates, the alloy’s microstructure transitions from being dominated by extensive dislocations and tangles to exhibiting characteristics of dynamic recovery and recrystallization. The dynamic recrystallization grains of the alloy achieve nucleation through the rearrangement and evolution of dislocations, gradually transforming subgrain boundaries into high-angle grain boundaries, which is continuous dynamic recrystallization [29].

## 4. Conclusions

(1) The flow stress of 7085 aluminum alloy exhibits a strong dependency on deformation conditions, diminishing as temperature rises and strain rate falls. The true stress–strain curves of the alloy generally show DRV characteristics, but it is also accompanied by DRX softening at high temperature and low strain rate. The strain-compensated Zener–Hollomon model established for the flow stress of 7085 aluminum alloy has a good predictive ability with a high R^2^ value of 0.992, a low ARRE value of 5.57%, and a low RMSE value of 4.38 MPa.
(2) Based on the DMM processing maps of 7085 aluminum alloy, the instability regions are distributed at the high-strain-rate zones with lower power dissipation efficiency (0.13–0.28). The optimal processing window region of 7085 aluminum alloy is 693–743 K/0.01–0.001 s^−1^, in which power dissipation efficiency is higher than 0.3.
(3) Compressed at low temperatures and high strain rates, the grains of the alloy are filled with dislocations and accompanied by a small amount of dislocation cells and subgrains formed via DRV. When temperature increases, and strain rate decreases, the dislocation density decreases, the number of dislocation cells and subgrains increases, and even a large number of serrated grain boundaries appear due to local and non-uniform grain boundary migration. Compressed at high temperatures and low strain rates, dynamic softening involves not only DRV but also continuous DRX.

## Figures and Tables

**Table 1:** The fitted coefficients for the 9th-order polynomial in Equation (8).

Coefficient	*α*	*n*	Q	*lnA*
B_0_	0.02	4.79	98.48	13.88
B_1_	0.16	−23.12	1176.43	206.36
B_2_	−3.10	389.33	−18,927.67	−3341.41
B_3_	29.54	−3653.54	158,880.42	28,442.74
B_4_	−162.21	201.74	−793,937.99	−145,441.79
B_5_	541.74	−68,285.97	2,476,519.81	467,911.79
B_6_	−1116.61	143,150.45	−4,871,870.16	−954,479.78
B_7_	1386.30	−180,948.51	5,881,244.24	1,197,597.26
B_8_	−949.96	126,196.81	−3,981,392.84	−842,318.18
B_9_	275.89	−37,262.87	1,157,578.84	253,827.69

## Data Availability

The original contributions presented in this study are included in the article. Further inquiries can be directed to the corresponding author.

## References

[1] Peterson L.A., Horstemeyer M.F., Lacy T.E., Moser R.D. (2020). Experimental characterization and constitutive modeling of an aluminum 7085-T711 alloy under large deformations at varying strain rates, stress states, and temperatures. Mech. Mater..

[2] Mei R.B., Du Y.X., Bao L., Zhang X.Y., Zhang B., Zhou Z.J. (2015). Study on hot deformation behavior of 7085 aluminum alloy during backward extrusion process. Model. Simul. Eng..

[3] Cao P., Li C., Zhu D., Zhao C., Xiao B., Xie G. (2023). Effect of Grain Structure and Quenching Rate on the Susceptibility to Exfoliation Corrosion in 7085 Alloy. Materials.

[4] Li S.S., Yue X., Li Q.Y., Peng H.L., Dong B.X., Liu T.S., Yang H.Y., Fan J., Shu S.L., Qiu F. (2023). Development and applications of aluminum alloys for aerospace industry. J. Mater. Res. Technol..

[5] Xie F., Yang Y., Ping Z., Gong X., Dong Z., Xu W.T., Yu J.Y., Yao W., Dong Y.Z. (2025). Progress and strategies in advanced aerospace materials for extreme environments: A review. Acta Astronaut..

[6] Li Y.J., Chen Y.Y., Zhao X.D., Qin F.M., Liang S.S. (2025). Hot deformation behavior and microstructural evolution of Sc and Zr micro-alloyed Al-Zn-Mg-Cu alloy. Mater. Charact..

[7] Deng Y., Zhao H., Wang X., Cui M., Zhao X., Zhang J., Zhou J. (2025). Microstructure Evolution and Constitutive Model of Spray-Formed 7055 Forging Aluminum Alloy. Materials.

[8] Malik A., Nazeer F., Al-Sehemi A.G. (2024). Constitutive description, processing maps, and un-explored deformation mechanisms of pure Mg and Mg-6Al(wt%) alloy under hot compression. J. Alloys Compd..

[9] Zhou H.T., Kong F.T., Wang X.P., Chen Y.Y. (2017). Hot deformation behavior and microstructural evolution of as-forged Ti-44Al-8Nb-(W, B, Y) alloy with nearly lamellar microstructure. Intermetallics.

[10] Mohammadi H., Eivani A.R., Seyedein S.H., Ghosh H.R.M., Jafarian H.R. (2021). An investigation of hot deformation behavior of Zn-22Al alloy and development of its processing maps during isothermal compression. J. Mater. Res. Technol..

[11] Rajput S.K., Chaudhari G.P., Nath S.K. (2016). Characterization of hot deformation behavior of a low carbon steel using processing maps, constitutive equations and Zener-Hollomon parameter. J. Mater. Process. Technol..

[12] Mei R.B., Zhang B., Cai B., Zhang X.Y., Zou Z.T., Liu Y.N., Li C.S. (2015). Study on constitutive equation of 7085 aluminum alloy under high deformation temperature. Adv. Mater. Res..

[13] Zhang Z., Liu R., Li D., Peng Y., Zhou G., Jia Z., Ma W. (2024). Investigation on deformation behaviors and dynamic recrystallization mechanism of spray formed Al-Zn-Mg-Cu alloy under hot compression. J. Mater. Res. Technol..

[14] Dai Q., Deng Y., Tang J., Wang Y. (2019). Deformation characteristics and strain-compensated constitutive equation for AA5083 aluminum alloy under hot compression. Trans. Nonferrous Met. Soc. China.

[15] Harun B., Kumar R., Jaiswal S., Huang E.W., Chang Y.J., Yeh A., Singh S.S., Neelakantan S., Jain J. (2026). Hot deformation behavior and processing map development of Al0.3Co1.5CrFeNi1.5Ti0.2 high-entropy alloy: Mechanisms and microstructural evolution. Intermetallics.

[16] Kareem S.A., Anaele J.U., Aikulola E.O., Olanrewaju O.F., Omiyale B.O., Falana S.O., Oke S.R., Bodunrin M.O. (2025). Hot deformation behavior of aluminum alloys: A comprehensive review on deformation mechanism, processing maps analysis and constitutive model description. Mater. Today Commun..

[17] Xiao W.C., Wang B.Y., Wu Y., Yang X.M. (2018). Constitutive modeling of flow behavior and microstructure evolution of AA7075 in hot tensile deformation. Mater. Sci. Eng. A.

[18] Park S.Y., Kim W.J. (2016). Difference in the hot compressive behavior and processing maps between the as-cast and homogenized Al-Zn-Mg-Cu (7075) alloys. J. Mater. Sci. Technol..

[19] Kwak T.Y., Lim H.K., Kim W.J. (2015). Hot compression characteristics and processing maps of a cast Mg-9.5Zn-2.0Y alloy with icosahedral quasicrystalline phase. J. Alloys Compd..

[20] Chen P., Zhang X., Chen D., Cao S., Jia Y., Zhang S., Han J. (2025). Dynamic recrystallization-based hot processing map and dynamic softening mechanism of Ti_3_Al alloy in wide strain rate ranges. Mater. Sci. Eng. A.

[21] Odeshi A.G., Tiamiyu A.A., Das D., Katwal N., Oguocha I.N.A., Khan A.K. (2019). High strain-rate deformation of T8-tempered, cryo-rolled and ultrafine grained AA 2099 aluminum alloy. Mater. Sci. Eng. A.

[22] Ke B., Ye L., Tang J., Zhang Y., Liu S., Lin H., Liu X. (2020). Hot deformation behavior and 3D processing maps of AA7020 aluminum alloy. J. Alloys Compd..

[23] Liu L., Zhao G., Wang G., Ma X., Yan Z., Cao S. (2023). Hot deformation behavior and microstructure evolution model of 7055 aluminum alloy. J. Mater. Res. Technol..

[24] Yang Q., Deng Z., Zhang Z., Liu Q., Jia Z., Huang G. (2016). Effects of strain rate on flow stress behavior and dynamic recrystallization mechanism of Al-Zn-Mg-Cu aluminum alloy during hot deformation. Mater. Sci. Eng. A.

[25] Dong F.L., Zhang D.Z., Sheng D.L., Zhao J.S., Zhang X.M., Wang Q., Su Q.H. (2016). Dynamic recrystallization behavior of 7085 aluminum alloy during hot deformation. Trans. Nonferrous Met. Soc. China.

[26] Zamani M., Dini H., Svoboda A., Lindgren L.E., Seifeddine S., Andersson N.E., Jarfors A.E. (2017). A dislocation density based constitutive model for as-cast Al-Si alloys: Effect of temperature and microstructure. Int. J. Mech. Sci..

[27] Rong Z.Z., Wu X.L., He F.Y., Wen S.P., Xiong X.Y., Gao K.Y., Wei W., Huang H., Nie Z.R. (2025). Hot deformation behavior, dynamic recrystallization and precipitation behavior of a novel Er, Zr-microalloyed Al-Cu-Mg alloy. J. Mater. Res. Technol..

[28] Zhang Z., Cong H., Yin Z., Qi B., Dong Y., Kong L., Prashanth K.G. (2024). Thermal deformation behavior and microstructural evolution of the rapidly-solidified Al-Zn-Mg-Cu alloy in hot isostatic pressing state. J. Mater. Res. Technol..

[29] Hong L., Wu X.L., Xiong X.Y., Gao K.Y., Wen S.P., Wei W., Rong L., Huang H., Nie Z.R., Dong Y. (2025). Study of dynamic recrystallization behavior of Al-Zn-Mg-Cu-Er-Zr alloy during isothermal compression. J. Mater. Res. Technol..

